# Lessons from COVID-19 Pandemic: A Successful Policy and Practice by Pasteur Institute of Iran

**DOI:** 10.61186/ibj.3964

**Published:** 2023-10-11

**Authors:** Mona Sadat Larijani, Alireza Biglari, Rahim Sorouri, Mostafa Salehi-Vaziri, Delaram Doroud, Keyhan Azadmanesh, Fatemeh Fotouhi, Ehsan Mostafavi, Amitis Ramezani

**Affiliations:** 1 Clinical Research Department, Pasteur Institute of Iran, Tehran, Iran;; 2 School of Medicine, Tehran University of Medical Sciences, Tehran, Iran;; 3 IPI Directorate, Pasteur Institute of Iran, Tehran, Iran;; 4 COVID-19 National Reference Laboratory, Pasteur Institute of Iran, Tehran, Iran;; 5 Quality Control Department, Production and research Complex, Pasteur Institute of Iran, Tehran, Iran;; 6 Department of Molecular Virology, Pasteur Institute of Iran, Tehran, Iran;; 7 Department of Influenza and Other Respiratory Viruses, Pasteur Institute of Iran, Tehran, Iran;; 8 Department of Epidemiology and Biostatistics, Research Centre for Emerging and Reemerging Infectious Diseases, Pasteur Institute of Iran, Tehran, Iran

**Keywords:** COVID-19, Pandemics, Vaccines

## Abstract

The present study aims to provide an insight to the comprehensive efforts of PII regarding COVID-19 management, research, achievements, and vaccine production, though there are many challenges. The relevant literature review was investigated through national and international database and also reports from the related research departments. Six strategies were taken by PII to manage the pandemic of COVID-19. While this pandemic has been hopefully controlled, SARS-CoV-2 could still be a potential threat. Therefore, COVID-19 data management and updated studies, as well as long-term safety and efficacy of the SARS-CoV-2 vaccines are still on the agenda.

## A FLASH BACK to the GLORIOUS HISTORY and ACTIVITIES of PII

PII, as one of the oldest leading public health and research centers in the country and the Middle East, was established in 1920 after an agreement between the Iranian government and Pasteur Institute of Paris. PII, a member of the global Pasteur network members, is among the few Pasteur Institutes capable of producing vaccines. This Institute has played a crucial role in the prevention and management of infectious diseases through conducting numerous types of research designs, manufacturing vaccines and producing biological products, as well as public health surveillance since its foundation^[^^1^^,^^2^^]^. Research departments at PII have been run to meet and suit the community’s health gaps during a-hundred-year of research work. Crucial steps have been taken to generate biopharmaceuticals through recombinant DNA technologies at the Institute since 1970s. There is also a specified building for biotechnology research group with the name of Dr. Marcel Baltazard, the former French director of the Institute, in honor of his great efforts in Iran^[^^3^^]^. 

PII conducts research projects by six scientific groups. These groups include 21 research departments with the pursued policy of basic and applied research, diagnosis, disease management, as well as via joint research projects with other centers^[^^2^^,^^4^^]^. Due to the growing urgent need for vaccination, recombinant products, and injectable solutions, Karaj Production Complex of PII started its mission in 1988. As a leading center for the human vaccine production in Iran, the Complex has provided PII with infrastructure and expanding activities. During the past century, PII has shown the power to control many infectious diseases, such as smallpox, plague, cholera, tuberculosis, rabies, hepatitis B, and COVID-19, by vaccination and effective interventions^[^^5^^-^^7^^]^. Moreover, as the national reference center for infectious diseases in the country, PII conducts diagnostic tests and research projects to promote the community health based on the public health priorities^[^^8^^]^. There are also other branches of the Institute with the aim of diagnosis and control of infectious diseases. A research and diagnostic center in Akanlu, (Hamadan Province) was founded in 1952, and Amol research branch in the city of Amol, northern Iran, was established in 1994^[^^9^^,^^10^^]^. 

## COVID-19 era

Iran with a population of about 84 million and a wide variety of cultures, socioeconomic conditions, and climate conditions has faced many hardships regarding the equipment and facilities. Thus, challenges imposed by the COVID-19 pandemic in the country differ from other countries^[^^11^^]^. During the early episode of COVID-19 era^[^^12^^]^, PII provided people with a rapid response team, which was established before the pandemic in Iran. This service was a help to overcome the stressful situation that the new virus imposed on the society. The first COVID-19 diagnostic test was then developed by this team, leading to the establishment of the first diagnostic laboratory for SARS-CoV-2 infection. All the collected COVID-19 suspected samples in the country were sent to this laboratory for diagnosis. The educated staff and experts put all their effort day and night to handle the tests immediately in order to prevent any potential crisis^[^^13^^,^^14^^]^. In addition to be the national COVID-19 reference laboratory, PII is now a center of excellence for SARS-CoV-2 diagnosis, epidemiologic studies, emerging and re-emerging infectious diseases, clinical trials, and notably vaccine manufacturing and research. Hence, we have presented successful strategies during the recent pandemic performed in PII, as the national COVID-19 laboratory committee. 

## PRACTICAL METHODS FOR MANAGEMENT of COVID-19 CHAOS

**COVID-19 laboratory network establishment**

**Fig. 1:**
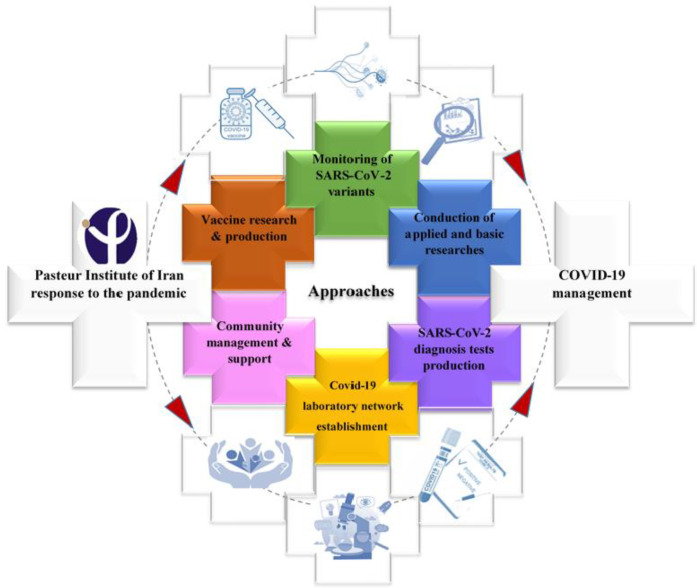
The map of success achieved by PII in the management of COVID-19 pandemic. In the Figure, there are six strategies by which the Institute managed the pandemic. These actions have been taken in parallel to obtain the best results

PII has contributed to the COVID-19 pandemic through six strategies presented in Figure 1. Although the COVID-19 pandemic imposed many hardships on Iran, great achievements, including COVID-19 laboratory network foundation, have been made. Following the identification of the novel coronavirus, the spread of forthcoming infection became a priority, which was managed by the experienced scientists at PII. The rapid response team of the Institute, which is authorized for the outbreak investigation/control and early diagnosis of infectious diseases, took the initial steps of establishing the first diagnostic laboratory to conduct the molecular tests.

In February 2020, the first COVID-19 laboratory was established at PII when the commercial kits had not yet been imported to Iran. In this regard, the Institute was authorized to establish the COVID-19 laboratory diagnostic committee and direct national laboratory diagnostic network with the support of the Ministry of Health^[^^13^^]^. Thousands of suspected COVID-19 samples were sent daily to PII after the recognition of the first case in the country on February 18, 2020. Nevertheless, the broad spread of the virus throughout the country led to the establishment of laboratories in each city, which later resulted in a well-grown number of 500 active labs with the capacity to test more than 100,000 cases per day^[^^15^^]^. PII also provided the candidate labs with molecular kits besides the staff training, evaluation of received PCR files and running the scheduled meetings with the lab team to check the quality of procedures in parallel with external quality control panels. PII undertook the verification of the imported COVID-19 kits since the beginning of its pandemic. After the initial evaluation of companies imported the kits, PII investigated the quality assessment of the diagnostic and diagnostic and immunological kits produced in the country. After about six months, the provided protocols and responsibilities were handed over to the Food and Drug Reference Laboratory^[^^16^^,^^17^^]^. Moreover, a specified group was created on the social network application in order to provide an easy and quick cyberspace, where the questions, requests, complaints and experiences of the laboratories are shared and responded.

**Community management and support**

The management of community health was achieved through remote and on-site policies. We specified landlines and cell phone lines to manage the outpatients. Following the phone contacts, the cases were given advice by an infectious disease specialist. Only the suspicious subjects who presented SARS-CoV-2 symptoms were advised to visit a doctor. Therefore, unnecessary referrals to the hospitals was significantly decreased. In addition, a specific place was considered to visit the suspected people for COVID-19 for sampling and diagnosis. Via specified phone lines, people were able to ask their questions and get precise recommendations from specialists regarding COVID-19. They were advised to refer to a medical center or specific hospitals only in case of necessity. This effort, which might seem simple at the first glance, had a significant impact on patients’ control; moreover, healthcare workers could do their tasks in a less-stressful atmosphere. Indeed, the remote guide of people resulted in a managed hospital referral system and avoided viral 

spread from the crowd to the medical centers. PII, as a trustful scientific center among the community, has also provided people with physicians and infectious disease specialists. The suspected individuals who presented COVID-19 symptoms could be visited in the Corona Virus Department. The naso/oropharyngeal swab samples were taken to go through the molecular test following specialists’ recommendation, and in case of a positive test, they could benefit from appropriate prescription and recommendation. Furthermore, educational and informative booklets and brochures were designed and published in the Institute to provide people with varying information on SARS-CoV-2 symptoms, transmission, prevention, and treatment.

**Monitoring SARS-CoV-2 variants**

The detection of SARS-CoV-2 variants at the right time was the main priority of PII’s agenda. Consequently, the main variants of SARS-CoV-2, including alpha, beta, delta, and omicron, were identified by PII using the molecular test (SANGER) prior to the severe peak of the new strain, with the least missing time. The approved results were sent to the member laboratories of the network. Monitoring SARS-CoV-2 genetic variation is currently conducted more effectively by applying next-generation sequencing and new sequencing devices. This attempt owes to the round-the-clock efforts of a large eager group of scientists, indicating the existence of admirable technical knowledge and a sense of responsibility across the country^[^^18^^]^. 

**Production of SARS-CoV-2 diagnostic test kits**

Owing to the fact that influenza and SARS-CoV-2 share multiple similarities in symptoms, and the rate of infected people with flu increases in cold weather, a standard test to detect these viruses was simultaneously developed. Pasteur COVI/FLU A&B was produced in the national COVID-19 center at PII to distinguish influenza type A/B and SARS-CoV-2, concurrently. The kit was first launched in November 2021 due to the probable increasing number of flu infections in fall and winter. This test is capable to recognize two conserved regions of SARS-CoV-2 nucleocapsid, one from influenza A matrix and one from influenza B nucleocapsid, on the extracted viral RNA from nasopharyngeal swab samples based on probe hydrolyzation. Pasteur Spike Gene Target Failure Real-Time PCR was also produced since the new variant of SARS-CoV-2, omicron, started spreading globally. The test was developed based on a one-step real-time RT-PCR to distinguish omicron variant from other SARS-CoV-2 types based on the two deleted parts of spike gene. 

**Conduction of applied and basic research**

A wide variety of SARS-CoV-2 studies have been conducted in PII. More than 100 manuscripts are accessible in NCBI PubMed database (https://pubmed. ncbi.nlm.nih.gov/) in which the Institute has contributed to SARS-CoV-2 publications, including basic, preclinical and clinical studies^[^^19^^-^^22^^]^, bioinformatics and in silico studies^[^^23^^-^^25^^]^, case reports^[^^26^^,^^27^^]^, reviews, and meta-analysis^[^^18^^,^^28^^]^, as well as publications on COVID-19 mechanisms^[^^29^^,^^30^^]^, long COVID^[^^31^^,^^32^^]^, and COVID-19 diagnosis and therapeutic targets^[^^33^^-^^36^^]^. However, only a few of these publications have been cited in this manuscript.

**Vaccine research and production**

During the COVID-19 pandemic, PII has manufactured PastoCovac (Soberana 02) and PastoCovac Plus (Soberana Plus) in collaboration with Finlay Institute of Cuba. The cooperation between the two countries started since hepatitis B vaccine technology transfer. PastoCovac and PastoCovac Plus technology were successfully transferred to Iran. PastoCovac is composed of a highly immunogenic part of SARS-CoV-2 spike (RBD), which has been conjugated to the tetanus toxin^[^^37^^]^. PastoCovac Plus, as the booster dose, is a dimer of SARS-CoV-2 spike (RBD)^[^^37^^-^^39^^]^. The conjugated COVID-19 vaccine successfully passed phase I and II trials in Cuba with significant results. Phase III clinical trial investigated in Iran and Cuba showed remarkable protection against the infection development, and the vaccine was hugely effective against the severe form of COVID-19 and death in the heterologous three-dose schedule^[^^37^^]^. Later, PastoCovac vaccine was evaluated in terms of safety and immunogenicity, in order to be administrated as a booster dose in Iran. The clinical trial phase III was conducted in the country in eight cities, and immunization by a three-dose regimen was significantly effective against hospitalization in the Delta variant era. PastoCovac was approved to be administrated to children above three years old, and at the same time, has been evaluated as an effective and safe booster vaccine for all primary vaccines in Iran^[^^40^^,^^41^^]^. In several studies carried out by PII (under review data), PastoCovac Plus immunogenicity was assessed among the individuals primarily immunized by two doses of Covaxin (BBV152), ChAdOx1-S, or BBIBP-CorV. PastoCovac Plus administration significantly led to anti-SARS-CoV-2-specific antibodies rise in the investigated groups with no serious adverse event^[^^42^^]^. According to the obtained data on a selected sample of health care workers who were primarily immunized with Covaxin, SARS-CoV-2 neutralization and anti-spike antibodies reached 70- and 93-fold-rise after PastoCovac Plus booster shot, respectively^[^^43^^]^. Another study, which evaluated the heterologous or homologous regimens among individuals vaccinated with ChAdOx1-S or BBIBP-CorV, showed that applying PastoCovac Plus as a booster dose in heterologous regimens is more effective than the homologous regimens. Moreover, the highest rate of anti-spike IgG rise observed in ChAdOx1-S-/PastoCovac Plus®, followed by BBIBP-CorV/PastoCovac Plus, which indicated that the heterologous vaccine design against SARS-CoV-2 could bring excellent results^[^^42^^]^. The studied safety and immunogenicity of PastoCovac, as a booster dose, in 18-80 years old people in Iran showed great immunogenicity and long-term protection six months post injection^[^^44^^]^. The investigation of the long-term adverse events of COVID-19 vaccines in recipients of homologous vaccine regimen and combined regimens of Sinopharm/Plus and AstraZeneca/Plus in a period of 18 months showed the vaccine adverse events are rare^[^^45^^]^. What is more, administration of COVID-19 boosters induced persistent humoral immune responses in individuals with underlying diseases who were primarily primed with Sinopharm vaccine, in whom PastoCovac/Plus booster shots led to a higher fold increase in antibodies and lower adverse events^[^^46^^]^. Since the vaccine approval, about 15 million doses of COVID-19 vaccines have been produced and released to the health ministry of Iran. Moreover, a capacity of three million doses of vaccine production is currently provided monthly, and PII has the potency to export PastoCovac vaccine to other countries in future. In addition to the mentioned approved COVID-19 vaccines in Iran, another vaccine, based on an adenovirus-based platform, was developed successfully, and the preclinical studies conducted via highly regulated standards. PastoCoAd was assessed as a novel heterologous recombinant adenovirus. The results showed that this vaccine candidate can induce both humoral and cellular immune responses in animal models^[^^47^^]^. Moreover, there has been an exchange agreement between PII and Bharat Biotech of India regarding rotavirus vaccine since 2021 in order to protect young children against infection^[^^48^^]^. Besides, the technical knowledge exchange of manufacturing pneumococcal vaccine was agreed between PII and Cuba to prevent infections such as pneumonia, sepsis, and meningitis^[^^49^^]^.

## CONCLUSION

PII stood tall and proud during the COVID-19 era. The recent pandemic resulted in an excellent performance and unity of the scientists in Iran. There is no doubt that PII has significantly contributed to COVID-19 management through the COVID-19 laboratory network set-up, two approved vaccines in collaboration with Cuba and one vaccine candidate on the go. Nevertheless, there are some probable difficulties in future, including preparation for any probable COVID-19 trend, data management, and long-term safety and efficacy of the vaccines and supplies, which need to be considered. The significant contribution of the scientists of the Institute to other research centers and laboratories was of great value and had a significant impact on COVID-19 pandemic management in the country.

## DECLARATIONS

### Acknowledgments

We would like to thank all the staff of Pasteur Institute of Iran who eagerly contributed to COVID-19 pandemic control. No artificial intelligence was used in this study.

### Ethical approval

Not applicable.

### Consent to participate

Not applicable.

### Consent for publication

All authors reviewed the results and approved the final version of the manuscript.

### Authors’ contributions

MSL and AR: wrote the original draft and contributed to the data collection and manuscript revision; AB, RS and EM: contributed to the study design and data curation; MSV, DD, KA, FF and EM: provided literature review and revised the manuscript.

### Data availability

All relevant data can be found within the manuscript. 

### Competing interests

The authors declare that they have no competing interests. 

### Funding

This research received no specific grant from any funding agency in the public, commercial, or not-for-profit sectors. 

### Supplementary information

The online version does not contain supplementary material.
